# Editorial: The many faces of brain aging

**DOI:** 10.3389/fnagi.2022.1018238

**Published:** 2022-08-30

**Authors:** Luca Marsili, Marco Canevelli, Federico Rodriguez-Porcel

**Affiliations:** ^1^James J. and Joan A. Gardner Center for Parkinson's Disease and Movement Disorders, Department of Neurology, University of Cincinnati, Cincinnati, OH, United States; ^2^Department of Human Neuroscience, Sapienza University of Rome, Rome, Italy; ^3^Department of Neurology, Medical University of South Carolina, Charleston, SC, United States

**Keywords:** aging, neurodegeneration, Parkinson's disease, Alzheimer's disease, dementia

Brain aging is characterized by changes at all levels, from molecules to morphology, reflected by reduced brain size, altered vasculature, and declines in motor and cognitive features (Canevelli and Marsili, 2022). These changes may (e.g., the “*pathological aging*”) or may not (e.g., the “*physiological aging*”) impair daily living activities. Motor and cognitive impairment constitute the most common phenotypic expressions of brain aging. Both phenomena often exist in the same disease category, thus making it difficult to detect “pure” motor or cognitive conditions (Schirinzi et al., 2020). Cognitive disturbances often characterize movement disorders, and neurodegenerative dementias often exhibit the occurrence of movement disorders. Brain aging may be investigated through its motoric, cognitive, neurophysiological, neuroimaging, and biochemical aspects. The development of biomarkers and other indicators of aging trajectories in the future would help disentangle the differences between what is physiological and what is pathological brain aging and revise the sharp separation between what is motoric and what is cognitive.

This special issue was designed to explore brain aging phenomena across neurodegenerative diseases. Seven of the nine papers initially submitted to the journal by international researchers were considered suitable for publication after a thorough peer-review process. These included five original research articles, one brief research report, and one opinion. The following is a summary of the main results of each of these manuscripts.

Recently, the immune system's role in neurodegeneration has been intensely investigated to identify possible mechanisms of neurodegeneration and related biomarkers. Fleury et al., investigated the clinical correlates of plasmatic pan-neurotrophic pro-brain-derived neurotrophic factor (BDNF) receptor p75^NTR^ extracellular domain (ECD) levels in older adults without clinically manifested neurological disorders. The p75^NTR^ receptor binds neurotrophins and plays an important role in neuronal survival and apoptosis. Recently, abnormal plasmatic levels of ECD of p75^NTR^ have been reported in multiple neurodegenerative disorders (Jiao et al., 2015). Plasma p75^NTR^ ECD levels were positively correlated with inflammatory markers interleukin-6 and CD40 Ligand and were negatively correlated with the platelet activation marker P-selectin. However, there was no association between plasma p75^NTR^ ECD levels and cognitive performance. Pandey explored the role of antibodies directed against alpha-synuclein (α-syn) in neurodegeneration. Increased plasma, cerebrospinal fluid, and brain level of α-syn and their association to microglial cell activation, pro-inflammatory cytokines production, neurodegeneration, and cognitive deficits have been observed in aged humans (Goldman et al., 2018). However, the exact mechanism by which such α-syn may trigger neuroinflammation in aged humans is poorly defined. Alpha-syn, α-syn-reactive IgG autoantibodies, and Fc gamma receptors (FcγRs) function are strongly implicated in brain aging phenomena.

The association between hemodynamic parameters, particularly chronic high blood pressure, and accelerated cerebral aging are somewhat widely established (Gasecki et al., 2013). Zimmermann et al., examined the interactions between brain ^18^F-FDG PET metabolism and multiple hemodynamic parameters at different ages. They identified extensive associations between cerebral metabolism and hemodynamic parameters, indicating common aging mechanisms. Heart rate throughout adult life, systolic and pulse pressure parameters around middle age, and finally diastolic pressure parameters in older ages, were the parameters most extensively associated with brain metabolism, thus suggesting the possible therapeutic targets to counteract accelerated brain aging.

In cognitive research, a consolidated aging pattern consists of well-preserved vocabulary, general knowledge across the lifespan, and linear decline of fluid cognitive abilities starting in early adulthood (Salthouse, 2009). Multiple lines of evidence show that vocabulary and common knowledge do not only resist decline, but they tend to improve with aging (Park et al., 2002). Kljajevic has investigated whether the interpretation of proverbs in healthy subjects differs across the lifespan and if it could be associated with age-related fronto-temporal atrophy. More in detail, old adults (OA) and middle-aged adults (MA) performed better on proverb interpretation than young adults (YA). Nevertheless, OA and MA, when compared to YA, showed significantly more atrophy in frontal and temporal lobes. The authors concluded that the interpretation of proverbs is well-preserved with aging, despite considerable age-related cortical atrophy. Asci et al., investigated the decline of handwriting with aging in healthy subjects through machine-learning algorithms. Handwriting is a complex cognitive and motor skill consequential to activating an extensive brain network, and it is known to decline with aging. In the study, participants' handwriting was digitalized through smartphones. Then, specific algorithms were used to measure, compare, and classify the main handwriting features across different age ranges. The machine-learning algorithm could discriminate handwriting features between the different age ranges (OA, MA, and YA) with good accuracy. In conclusion, the effect of aging on handwriting abilities can be remotely and objectively detected through machine-learning algorithms.

Retinal imaging has reached interest in the aging population, to help in the early detection of brain disorders. The retina and the brain share similar embryogenic origin and vasculature features (Hart et al., 2016). Tao et al., explored Retinal microvasculature and imaging markers of brain frailty in healthy adults. By combining brain MRI and deep learning techniques of optical coherence tomography and correlating them with cognitive testing, they showed that macular microvascular changes might reflect the cerebral radiological indicators associated with brain frailty in aging individuals.

Finally, regarding studies on animal models, Ma et al., explored the afferent and efferent projections of the rostral anterior cingulate cortex in young and middle-aged mice. It is well-known that, across life, the incidence of mental illness is highest at young ages. The rostral anterior cingulate cortex (rACC) is essential in psychiatric disorders and related chronic pain-psychiatric comorbidities. However, it is still unknown whether or how the afferent and efferent circuits of the rACC may change with aging phenomena. This study scrupulously analyzed the input-output neural projections of rACC in mice of different ages and sexes, thus providing preliminary data for further targeted research.

In conclusion, the editors wish to thank all the authors, the reviewers, and the editorial board members for contributing to this Research Topic. We hope this Research Topic might inspire future and novel research approaches in the field of brain aging.

## Author contributions

All authors listed have made a substantial, direct, and intellectual contribution to the work and approved it for publication.

## Conflict of interest

The authors declare that the research was conducted in the absence of any commercial or financial relationships that could be construed as a potential conflict of interest.

## Publisher's note

All claims expressed in this article are solely those of the authors and do not necessarily represent those of their affiliated organizations, or those of the publisher, the editors and the reviewers. Any product that may be evaluated in this article, or claim that may be made by its manufacturer, is not guaranteed or endorsed by the publisher.

## References

[B1] Canevelli M. Marsili L. (2022). Ageing of the Brain. Pathy's Principles and Practice of Geriatric Medicine. Hoboken, NJ: John Wiley & Sons Ltd. 68–76. 10.1002/9781119484288.ch6

[B2] Gasecki D. Kwarciany M. Nyka W. Narkiewicz K. (2013). Hypertension, brain damage and cognitive decline. Curr. Hypertens. Rep. 15, 547–558. 10.1007/s11906-013-0398-4 24146223 PMC3838597

[B3] Goldman J. G. Andrews H. Amara A. Naito A. Alcalay R. N. Shaw L. M.. (2018). Cerebrospinal fluid, plasma, and saliva in the BioFIND study: relationships among biomarkers and Parkinson's disease Features. Mov. Disord. 33, 282–288. 10.1002/mds.27232 29205509 PMC5836918

[B4] Hart N. J. Koronyo Y. Black K. L. Koronyo-Hamaoui M. (2016). Ocular indicators of Alzheimer's: exploring disease in the retina. Acta Neuropathol. 132, 767–787. 10.1007/s00401-016-1613-6 27645291 PMC5106496

[B5] Jiao S. S. Bu X. L. Liu Y. H. Wang Q. H. Liu C. H. Yao X. Q.. (2015). Differential levels of p75NTR ectodomain in CSF and blood in patients with Alzheimer's disease: a novel diagnostic marker. Transl. Psychiatry. 5, e650. 10.1038/tp.2015.146 26440538 PMC4930124

[B6] Park D. C. Lautenschlager G. Hedden T. Davidson N. S. Smith A. D. Smith P. K.. (2002). Models of visuospatial and verbal memory across the adult life span. Psychol. Aging. 17, 299–320. 10.1037/0882-7974.17.2.299 12061414

[B7] Salthouse T. A. (2009). When does age-related cognitive decline begin? Neurobiol. Aging. 30, 507–514. 10.1016/j.neurobiolaging.2008.09.023 19231028 PMC2683339

[B8] Schirinzi T. Canevelli M. Suppa A. Bologna M. Marsili L. (2020). The continuum between neurodegeneration, brain plasticity, and movement: a critical appraisal. Rev. Neurosci. 31, 723–742. 10.1515/revneuro-2020-0011 32678804

